# Promoting Mental Health in Healthcare Workers in Hospitals Through Psychological Group Support With Eye Movement Desensitization and Reprocessing During COVID-19 Pandemic: An Observational Study

**DOI:** 10.3389/fpsyg.2021.794178

**Published:** 2022-01-27

**Authors:** Elisa Fogliato, Roberta Invernizzi, Giada Maslovaric, Isabel Fernandez, Vittorio Rigamonti, Antonio Lora, Enrico Frisone, Marco Pagani

**Affiliations:** ^1^Asst Lecco, Lecco, Italy; ^2^EMDR Italy Association, Varedo, Italy; ^3^Institute of Cognitive Sciences and Technologies, Consiglio Nazionale delle Ricerche, Rome, Italy

**Keywords:** COVID-19, HealthCare Workers (HCW), psychological support, EMDR-IGTP, observational study

## Abstract

**Background:**

Psychological support was provided by the Eye Movement Desensitization and Reprocessing Integrative Group Treatment Protocol (EMDR-IGTP) within the hospitals in the Northern Italy in favor of healthcare workers during the COVID-19 pandemic. This study aimed at evaluating the effectiveness of treatment in terms of (a) symptomatology reduction related to peri- and post-traumatic stress; (b) clinical improvement over time; and (c) the maintenance of the achieved outcome over time.

**Methods:**

The population was composed of healthcare workers who spontaneously requested psychological intervention in both the first and the second emergency waves. Statistical analyses were carried out to highlight the differences in Impact of Event-Revised (IES-R) and Post-Traumatic Growth Inventory (PTGI) before and after the group intervention.

**Results:**

In both the first and the second waves, pre-treatment values are higher than post-treatment values for all dimensions of the IES-R. The results show that there are no significant differences between the first and the second wave with regard to the treatment effect. Healthcare workers maintained positive changes over time despite their prolonged exposure to an emergency and the possibility of retraumatization at the onset of a new emergency phase, irrespective of their working place. Healthcare workers who were treated in the first wave showed at the beginning of the second emergency wave less vulnerability and more resilience than those who were treated only in the second wave.

Pre-treatment scores of healthcare workers affected by COVID-19 are discussed.

**Conclusion:**

COVID-19 had a significant impact on the well-being of healthcare workers who were working in hospitals. Psychological support in case of emergency is needed.

## Introduction

In early 2020, when the issue of public health emergency due to a new pandemic outbreak (COVID-19) (World Health Organization, 2020a,b) was faced by communities and services, there was a reorganization of hospital facilities aiming at containing and managing the pandemic emergency. Healthcare facilities, from wards to hospital outpatient services and administrative offices, have undergone a significant structural, organizational, and care transformation. Specifically, the COVID-19 emergency brought difficulties in implementing the usual strategies for managing the problems at the organizational, structural, and individual level, making it necessary to draw up reports containing operational guidelines for the prevention of stress in healthcare and social care workers and the preparation of interventions aiming not only at their physical self-care but also at psychological self-care (ISSS, 2020).

The most significant changes concerned were the professional and personal lives of healthcare workers (World Health Organization, 2020c) and of all workers who, in hospital settings, found themselves working in contexts of uncertainty, powerlessness, and the lack of control; and the conditions that were aggravated by exposure to the risk of infection for themselves and their families and colleagues and that could be induced with a high probability of the onset of burnout (Giusti et al., 2020; Liu et al., 2020; Ornell et al., 2020; Ran et al., 2020). They were more exposed to biological risks and experienced a significant concern to their own health and the fear of spreading the infection to their family members, thus experiencing in many cases the fatigue of social distancing from their loved ones and, in particular, their children. The critical events that characterized the COVID-19 emergency left healthcare workers exposed to a high level of professional and personal stress, and they represented the risk factors for the development of traumatic stress reactions. In addition, the specific nature of this emergency, which occurred in the successive waves imposing the return to new epidemic waves, left workers exposed to a retraumatization. Having been exposed to direct and vicarious traumatization (Brady et al., 1999; Sinclair and Hamill, 2007) and being victims at multiple levels of an unprecedented emergency made all healthcare workers very vulnerable (Taylor and Frazer, 1981; Mitchell, 1983; Iacolino and Cervellione, 2019). This has been compounded by the risk of stigma (World Health Organization, 2020d).

Several cross-sectional studies through data collection measures in the form of online surveys or the administration of self-report scales (de Girolamo et al., 2020; Kang et al., 2020; Lai et al., 2020; Lu et al., 2020; Rossi et al., 2020; Santarone et al., 2020; Tran et al., 2020), as well as systematic reviews and meta-analysis (Giorgi et al., 2020; Luo et al., 2020; Xiong et al., 2020), reported the impact of COVID-19 on healthcare workers. These studies also highlighted the occurrence of anxiety, depression, distress, and Post-Traumatic Stress Disorder (PTSD) and underlined that being a frontline healthcare worker was an important risk factor for the development of severe psychological symptoms.

Due to the impact that this pandemic had on the mental health of healthcare workers, the protection of frontline responders in hospitals was an important component of public health measures to address COVID-19 emergency, considering the promotion of their mental health as a key element (Center for the Study of Traumatic Stress, 2020; Homewood Health, 2020; IASC, 2020; Liang et al., 2020; World Health Organization, 2020c). Therefore, from the outset, an important concern for those who were structuring psychological support interventions in hospitals aiming at health workers was to prevent the consequences of this pandemic on mental health (De Mei et al., 2020; Invernizzi et al., 2020a,b; Leone et al., 2020). Although several studies have recommended the need to provide specific psychological support to healthcare workers involved in the COVID-19 pandemic during and after the emergency, there are no data to analyze how their mental health conditions were taken care of and how the psychological interventions were structured in various hospital facilities and monitored in terms of the reduction of symptoms and clinical improvement over time.

This study was approved and authorized among the COVID-19 studies, by the Ethics Committee ATS Brianza with the acronym HOPE (Healthcare wOrkers Psichology Emdr) on March 19, 2021. This is a retrospective observational study since it measures the treatment conducted in a period prior to the design of the study (March 2020) and is prospective because it evaluates the effects of the intervention addressed to the personnel involved in the two waves of the pandemic to observe the outcomes and the efficacy of the intervention itself.

This study represents the evidence of an approach of mental health and psychosocial support (MHPSS) provided within the hospitals of the Azienda Socio Sanitaria Territoriale (Asst) of Lecco (Northern Italy) addressed to healthcare workers by psychologists belonging to the Simple Departmental Unit of Clinical Psychology of the same Asst. The composite term “MHPSS” is used in the Inter-Agency Standing Committee Guidelines for MHPSS in Emergency Settings (IASC, 2020) to describe any type of support that aims to treat a mental health condition. The global humanitarian system uses the term MHPSS to describe a broad range of stakeholders responding to emergencies, for instance, the COVID-19 outbreak, including those working in health settings, such as hospitals. In this perspective, since the beginning of the pandemic, the psychologists of the Asst of Lecco have shared the urgency to activate interventions for the treatment of healthcare workers as effective and evidence-based as possible, being aware that this health emergency represented a challenge to psychological resilience for all. The interventions activated were adapted to the needs of this specific population and the different phases of the COVID-19 pandemic. In March 2020, a COVID-19 Crisis Unit was formed by 20 psychologists, 14 of whom were qualified in the application of Eye Movement Desensitization and Reprocessing (EMDR, 2019) Therapy. Under the free supervision of the EMDR Italy Association, interventions have been activated at various levels, aiming at patients, healthcare workers, and social care workers suffering from COVID-19, and those working in hospitals.

Eye Movement Desensitization and Reprocessing therapy is recommended for the treatment of PTSD (International Society for Traumatic Stress Studies (ISTSS), 2018) and recognized by World Health Organization (2013) as an elective and advanced therapy in emergencies to manage specific stress-related disorders. A specific protocol of the group EMDR-Integrative Group Treatment Protocol (IGTP) approach (Jarero and Artigas, 2014) adapted to the COVID-19 emergency implies the use of bilateral self-stimulation represented by the butterfly hug (BH) (Artigas and Jarero, 2014). Pre- and post-treatment self-report scales with the group EMDR-IGTP were used to assess the reduction of stress-related symptomatology and perceived changes during time. In compliance with the distancing rules, the groups within the various hospital departments consisted in a minimum of two participants up to a maximum of four. Each group carried out three sessions in 1 month.

The primary outcome of this study was to evaluate the effectiveness of the treatment with EMDR-IGTP in the two emergency waves in terms of (a) a reduction in symptomatology related to peri- and post-traumatic stress and (b) clinical improvement over time. The secondary outcome was to evaluate the early treatment of healthcare and social care workers during a health emergency that occurs in phases to prevent an increased risk, at each subsequent phase, of developing traumatic stress reactions.

The decision to treat all healthcare workers promptly and early who have requested an intervention has responded to a professional and ethical choice of Healthcare in public health that considers healthcare workers as recipients of an immediate and urgent intervention of those working in a sanitary emergency, risking not only their physical health but also their psychological and mental health.

## Materials and Methods

### Setting

The population of the present study is composed of healthcare workers recruited from the two hospitals of the Asst of Lecco (A. Manzoni Hospital in Lecco and L. Mandic Hospital in Merate) who spontaneously requested psychological intervention in both the first (March to September 2020) and the second emergency waves (September 2020 to June 2021). Healthcare workers were treated with EMDR-IGTP (Jarero and Artigas, 2014) adapted to the COVID-19 emergency; three sessions were conducted in small groups of three or four participants in compliance with the rules of social distancing. Participation took place in respect of privacy and protection of sensitive data of the subjects involved as per the forms regarding the processing of personal data and informed consent.

### Subjects

A total of 360 healthcare workers were treated, 300 in the first emergency wave and 60 in the second wave. The subjects included in the HOPE study were: 107 healthcare workers treated in the first wave (10 men, 97 women, mean age 46.4 ± 8.5) and 43 treated in the second wave (5 men, 38 women, mean age 47.7 ± 9.4), for a total of 150 healthcare workers. The rest of the subjects (*n* = 210) could not be included since their questionnaires were incomplete or the post-treatment evaluations were not available or lacking.

**FIGURE 1:**
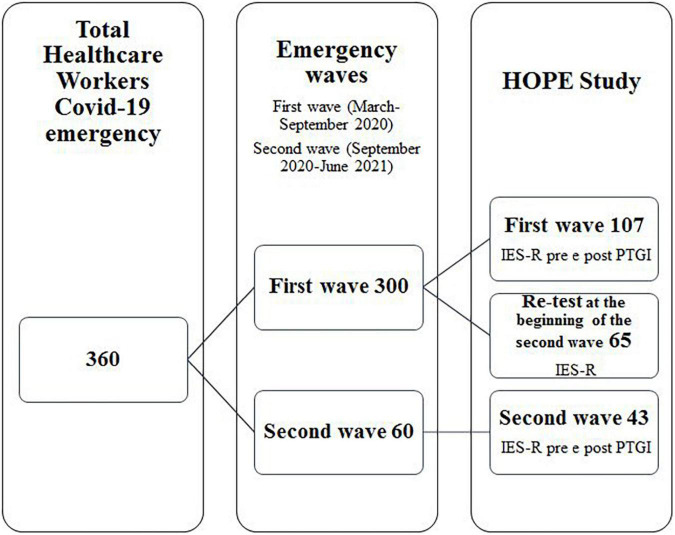
Research design.

From an ethical point of view, it was important that they received EMDR treatment even if their data were not complete to be included in the HOPE study. Regarding the healthcare workers who were treated in the first wave, 65 were re-tested at the beginning of the second emergency wave (refer to Figure 1).

Healthcare workers presented different professional profiles and high multiple problems. They were divided into three areas of origin:

I. Critical care area (intensive and sub-intensive care units);
II. COVID wards (converted COVID hospital wards);
III. Other (non-COVID departments, presidium medical directorates, administration, mortuary, and hospital chapel).

All healthcare workers (a) engaged in the emergency from March 2020 to June 2021 were included in the study protocol; (b) screened for the risk of developing PSTD assessed through the Impact of Event-Revised (IES-R) self-report scale administered pre- and post-treatment and the Post-Traumatic Growth Inventory (PTGI) post-traumatic growth questionnaire administered post-treatment; and (c) with properly completed documentation (registry and self-report scales).

### Assessment

Recruitment was voluntary. Group treatment with EMDR-IGTP focused on COVID-19-related trauma, and the aim to support them during the emergency was explained to all healthcare workers. In presence, treatment was proposed and shared to their departments, in small groups of three or four participants. The therapist who led the group was qualified in the use of EMDR. All EMDR therapists were supervised by an EMDR consultant.

For each subject, a personal data sheet was compiled, which included sociodemographic and work data (name, age, sex, educational qualification, professional qualification, and composition of the household) and individual events related to the COVID-19 emergency, such as the organization from the housing point of view during the health emergency (isolation and non-isolation), the presence of minor children and their placement during lockdown periods (if entrusted to others or not entrusted), having contracted the virus, family members infected or died from COVID-19, ward of origin before/during/post emergency, contacts with COVID-19 patients, working in a “dirty” or “clean” area, and recruitment in the different emergency waves.

The following self-report scales were administered before and after the group intervention:

*Impact of event scale-revised* (IES-R) was conducted with the purpose of measuring stress levels and symptomatology due to the impact of a traumatic pandemic event. The IES-R (Weiss and Marmar, 1997; Pietrantonio et al., 2003) is a self-report scale to measure psychological distress in response to a traumatic event. It consists of 22 items and includes 3 subscales (Intrusiveness, Avoidance, and Hyperarousal). Subjects were indicated on a Likert scale from 0 (not at all) to 4 points (very much) how frequently they have experienced each symptom in the past week. A total IES-R score equal to or greater than 33 represents the cutoff for the risk of developing PTSD. The IES-R was found to be highly internally consistent (Cronbach’s alpha, *a* = 0.96; Creamer et al., 2003).

*Post-Traumatic Growth Inventory* (PTGI) (Tedeschi and Calhoun, 1996; Prati and Pietrantoni, 2014) is a self-report questionnaire on post-traumatic growth to measure personal and interpersonal changes related to a pandemic event. The scale consists of 21 items with response mode on a Likert scale from 0 (no change) to 5 (very important change) and measures the positive outcomes reported by people who have faced negative and adverse experiences (Cormio et al., 2017).

### Treatment

#### Eye Movement Desensitization and Reprocessing

The intervention on frontline responders included three sessions. The duration of each session was 90 min. Each group consisted of a minimum of two healthcare workers up to a maximum of four for safety reasons. All interventions were conducted within the hospitals in a dedicated setting. The specific protocol of the group EMDR-IGTP approach (Jarero and Artigas, 2014) was adapted to the COVID-19 for a traumatic event (Jeffries and Davis, 2013; Carletto et al., 2017; Pagani et al., 2017). Emergencies with the use of bilateral self-stimulation were represented by the *BH* (Artigas and Jarero, 2014; Maslovaric, 2020). The use of an alternative bilateral stimulation (BH) appears to produce a physiological effect promoting adaptive reprocessing of dysfunctionally stored and related information.

The EMDR-IGTP administers the eight phases of the standard EMDR individual treatment (Shapiro, 1995, 2018) in a group format (Artigas et al., 2000; Jarero et al., 2006, 2008), using art therapy (i.e., drawings and symbols) and the BH, as a self-administered bilateral stimulation method to reprocess a traumatic material and has several advantages in an emergency (Pérez et al., 2020): it can be carried out in emergency settings; participants do not have to verbalize information about the trauma; therapy can be carried out in consecutive sessions; and more people can be treated at the same time (Maslovaric, 2020). Participants draw at the beginning a picture of the worst or representative part of the traumatic event, indicating the level of disturbance. They proceed with the self-administered bilateral stimulation (BH) and after some sets, they draw again what they notice as the processing occurs. In this case, the clinician can also follow the changes that each participant is having. They go on with the processing and they make another drawing in order to check the process. After some more sets, they do a final drawing with a positive image of themselves in the future. The adaptation of the protocol during the COVID-19 emergency specifically concerned the stabilization exercises in Phase 2 related to breathing: in fact, in the healthcare workers, it functioned as a trigger reactivating the traumatic experience related to the assistance of patients with COVID-19 in assisted ventilation or intubated. It was, therefore, important to use the technique of grounding instead of breathing exercises.

Eye Movement Desensitization and Reprocessing is a therapeutic approach used for the treatment of trauma and traumatic stress-related issues based on the Adaptive Information Processing (AIP) model (Shapiro, 2000). The aim of EMDR is to restore a natural way of processing the information in the memory to achieve an adaptive resolution through the creation of new, more functional connections. EMDR is considered to be one of the elective psychotherapeutic treatments for PTSD, according to several meta-analyses and clinical guidelines, and its neurobiological effects are also supported by neuroimaging findings (Shapiro, 2000; Pagani et al., 2012; Boccia et al., 2015; Carletto et al., 2018). Today it is recognized as an evidence-based method for the treatment of PTSDs (Feske, 1998; Bohart and Greenberg, 2002; Baek et al., 2019; Maddox et al., 2019) approved by the Ministero della Salute (2003), the American Psychiatric Association (2004), and the International Society for Traumatic Stress Studies (ISTSS) (2018). The World Health Organization (2013) recognized EMDR as an effective treatment for trauma and trauma-related disorders (Castelnuovo et al., 2019).

### Statistical Analysis

The data were extracted and then analyzed to detect their main statistical features and were processed by following these steps:

#### Reduction of Symptoms Related to Peri- and Post-traumatic Stress

The first statistical analysis refers to the pre- and post-treatment difference for the different IES-R and total IES-R scales (delta analysis). This corresponds to a paired data inferential approach focused on the difference from 0 (null hypothesis) to the difference between the before and after EMDR-IGTP conditions. A statistically significant difference is checked using both parametric (*t*-test) and non-parametric (Mann–Whitney’s U, signed-rank, and sign test) approaches. Considering, from the literature studies, the threshold of total IES-R ≥ 33 for the presence of risk indicators of structuring PTSD, the relative frequency of healthcare workers with total IES-R ≥ 33 in the two waves was calculated.

#### Clinical Improvement

The difference between the different subscales of the IES-R and total score measured at the end of a course of treatment with EMDR was analyzed in healthcare workers treated in the first wave and measured in the same group at the beginning of the second wave (re-test). From a statistical point of view, this is solved using a Student’s *t*-test (Mann–Whitney’s U in the case of deviation from the normal distribution) between the values of the IES-R in the two groups.

A comparison was also made between the IES-R of healthcare workers reassessed at the beginning of the second wave and the pre-treatment IES-R of healthcare workers encountered in the second emergency wave and not treated in the first (Student’s *t*-test). These (and next) results were obtained by performing the second-order statistics that analyze the differences in terms of “entry value” (pre-treatment) using a non-parametric test (Wilcoxon) based on the categorization by ranks; the *wave* variable is the variable that identifies the two groups of subjects and the different deltas are compared (delta analysis).

#### Maintenance of the Achieved Outcome

The experimental design simulates a dose-effect relationship study to compare the efficacy of EMDR in the group treated in the first wave (pre- and post-treatment assessment, delta first wave) with that of the group assessed in the same way at the beginning of the second wave, and then to reassess after the treatment with EMDR (delta second wave). A two-way ANOVA with a repeated factor (within the group: time) and a between-group (between) factor was then performed to observe a significant value of the time–group interaction.

Variables with zero variance or those that were found to be non-significant were eliminated from the analysis of the results of the present study (“number of household members,” “living situation during the emergency,” “relatives who died due to COVID-19,” and “wards they belonged to before and during the emergency”).

## Results

Table 1 lists the main data on sociodemographic, work, and clinical factors that contribute significantly to the discussion of the results. The sample is composed predominantly of female participants (first wave: F 90.7%; second wave: F 88.4%). Because of this imbalance, the “sex” covariate was not taken into account. The average age of healthcare workers was almost the same in both waves (46.5 vs. 47.7). Schooling in the first wave has a significant effect on baseline (total IES-R pre-treatment) but does not change the delta, i.e., the effect of EMDR-IGTP. Specifically, higher schooling corresponds to lower values of the total pre-treatment IES-R score. The effect of schooling, which is present in the first wave, is not noticeable in the second wave. This discrepancy is confirmed by the value of Spearman’s correlation coefficient between schooling and the total pre-treatment IES-R, which shows a significant decrease with schooling in the first wave (*F* = 4.89; *p* < 0.03) and no relevant relationship in the second wave. The percentage of healthcare workers who fell ill with COVID-19 increased from 14% (15/107) in the first wave to 32.6% (14/43) in the second wave. With respect to their professional role, there is a prevalence of nurses in both waves (79.4% in the first wave and 58.1% in the second wave) and a higher percentage value in the second wave for healthcare workers from places other than COVID wards (8.4% in the first and 25.6% in the second waves). However, the analyses show that the covariate “ward” does not have a significant effect neither on the pre-treatment condition (total IES-R) nor on the delta total IES-R pre- and post-treatment in both first and second waves.

**TABLE 1:** Significant sociodemographic, occupational, and clinical factors.

Variables	N 1st Wave	N 2nd Wave	% 1st Wave	% 2nd Wave	Variables	N 1st Wave	N 2nd Wave	% 1st Wave	% 2nd Wave
Sex					Positive to Covid				
M	10	5	9.3%	11.6%	YES	15	14	14.0%	32.6%
F	97	38	90.7%	88.4%	NO	92	29	86.0%	67.4%
Schooling					Occupation				
Elementary	0	0	0.0%	0.0%	Doctors	7	0	6.6%	0.0%
Lower middle schools	4	2	3.7%	4.7%	Nurses	85	25	79.4%	58.1%
Secondary schools	36	19	33.6%	44.2%	NA	6	7	5.6%	16.3%
Post-high school course	14	7	13.1%	16.3%	Other	9	11	8.4%	25.6%
Bachelor’s degree	24	9	22.4%	20.9%	Department				
Master’s degree	7	1	6.5%	2.3%	Critical area	58	12	54.2%	27.9%
Master	15	4	14.0%	9.3%	Covid Department	40	11	37.4%	25.6%
Post-graduate, PhD	7	1	6.5%	2.3%	Other	9	20	8.4%	46.5%
Children < 18									
Yes	43	12	40.2%	27.9%					
No	64	31	59.8%	72.1%					

*N, number; M, men; F, women; NA, nursing assistant; and Other, healthcare workers from places other than coronavirus disease (COVID) wards.*

As for the symptomatology reduction following EMDR-IGTP, we observed a significant reduction in the scores of all IES-R subscales (avoidance, intrusiveness, and hyperarousal; *p* < 0.001) resulting in a significant decrease of the total scores from 45.45 pre-treatment to 31.13 post-treatment (*p* < 0.001, Table 2). This also held true in the second wave in which the single subscales and the total scores (50.21 vs. 34.37) decreased significantly (*p* < 0.001; Table 2).

**TABLE 2:** Impact of Event-Revised (IES-R) differences between before pre- and post-treatment.

IES-R first wave	M pre-	SD pre-	M post-	SD post-
Avoidance	15.16	5.90	11.48	7.00
Intrusiveness	17.66	6.71	11.37	6.00
Hyperarousal	12.82	5.33	8.27	5.00
Total	45.65	15.83	31.13	16.21
**IES-R 2nd wave**	**M pre**-	**SD pre**-	**M post**-	**SD post**-
Avoidance	17.02	7.34	12.14	7.22
Intrusiveness	18.12	7.04	12.58	6.88
Hyperarousal	15.07	5.12	9.65	5.52
Total	50.21	16.99	34.37	17.15

*Differences in all variables before vs. after reached the statistical significance at p < 0.001. M, mean; SD, standard deviation.*

When analyzing the differences in terms of “entry values” (pre-treatment scores in the first and the second wave), the second wave showed higher mean values, where the differences reach statistical significance for all indices of the IES-R (*p* < 0.001), speaking in favor of greater severity of the second wave as compared to the first one.

When comparing the differences in the delta between pre- and post-treatment in the two waves, we found no significant difference in terms of total EMDR-IGTP scores (14.52 vs. 15.84, respectively, refer to Table 3).

**TABLE 3:** Delta of IES-R variables in the first and second waves.

IES-R	MΔ 1st Wave	SD	MΔ 2nd Wave	SD
ΔAvoidance	3.68	5.47	4.88	8.11
ΔIntrusiveness	6.29	6.27	5.53	7.22
ΔHyperarousal	4.55	4.41	5.42	6.22
ΔTotal	14.52	13.00	15.84	19.25

*M, mean; SD, standard deviation.*

The cutoff of the IES-R score for the risk of developing PTSD is considered to be equal to or greater than 33. In our study, the percentage of healthcare workers investigated in the first wave and bearing such a risk of PTSD was 80% in pre-treatment, decreasing to 40.91% in post-treatment. In the second wave, the investigated subjects at risk were 83.72% in pre-treatment and 55.81% in post-treatment (Table 4). This can be also inferred by Table 2 in which the scores relative to IES-R decreased in the first wave from about 46 ± 16 to 31 ± 16 and in the second wave from about 50 ± 17 to 34 ± 17.

**TABLE 4:** Analysis of the risk for Post-Traumatic Stress Disorder (PTSD) pre- and post-treatment in the two emergency waves.

PTSD first wave	M	SD
% HCW at risk of PTSD pre-treatment	80.00%	0.40
% HCW at risk of PTSD after treatment	40.91%	0.49
**PTSD 2nd wave**		
% HCW at risk of PTSD pre-treatment	83.72%	0.37
% HCW at risk of PTSD after treatment	55.81%	0.50

*HCW, healthcare workers; M, mean; SD, standard deviation.*

**FIGURE 2:**
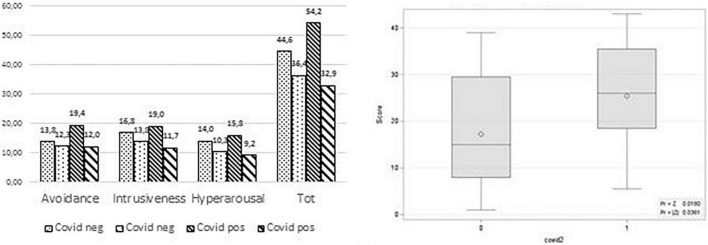
**Left:** Second wave HCW positive and negative to COVID pre-post treatment scores. **Right:** Wilconox score distribution of subscale avoidance HCW pre-post treatment COVID positive.

For the first wave, while analyzing the data of healthcare workers who got infected with COVID-19 (COVID positive) and those who did not get infected (COVID negative), no difference emerged with regard to deltas (the EMDR effect). However, significant differences were found in the second wave between pre- and post-treatment when comparing healthcare workers with COVID positive to those with COVID negative. The latter showed a less marked improvement than the positives in all scores in which the total IES-R scores (about 54.24 vs. 36.39; *p* < 0.05) and the subscale avoidance scores (19.36 vs. 12.04; *p* < 0.05) reached statistically significant differences (Table 5). As for the absolute values (pre- and post-treatment scores of the subscales considered separately), all the pre-treatment variables were higher in the COVID positive healthcare workers (Figure 2) even if only a comparison between the scores of the subscale avoidance reached statistical significance (19.36 vs. 13.78; *p* < 0.01). When the post-treatment total IES-R scores of 65 healthcare workers treated in the first wave were compared with the same scores at the beginning of the second emergency wave, no differences were found (31.13 vs. 30.17; Table 6). Notably, both average scores did not reach the sub-threshold value for the risk of developing PTSD, decreasing further several months after the conclusion of the treatment with EMDR-IGTP.

**TABLE 5:** Second wave HCW positive and negative to COVID.

	Negative		Positive	
IES-R 2nd wave	M pre	M post	M pre	M post
Avoidance	13.78	12.28	**19.36**	**12.04** ***
Intrusiveness	16.83	13.83	19.04	11.68
Hyperarousal	14.00	10.28	15.84	9.20
Total	44.61	36.39	**54.24**	**32.92** ***

HCW, healthcare workers. The comparison reached the statistical significance at ***p < 0.05. M, Mean was shown in bold. Pre- and post-treatment IES-R scores.

**TABLE 6:** A difference between total IES-R post-treatment in the first wave and total IES-R at retest.

IES-R	M	SD
IES TOT post 1st wave	31.13	16.21
IES TOT retest begin second wave	30.17	17.81

*M, mean; SD, standard deviation.*

Post-Traumatic Growth Inventory variables do not show a significant difference between the two waves.

## Discussion

The main aim of this study was to assess the effectiveness of treatment with EMDR-IGTP addressed to healthcare workers during the first and second wave of COVID-19 emergency in terms of (a) the reduction of symptoms related to traumatic stress reactions and (b) clinical improvement over time. The secondary objective was to assess the importance of treating healthcare workers early during the COVID-19 pandemic waves to prevent the increased risk, at each subsequent phase, of developing traumatic stress reactions. Our sample included predominantly women, confirming their dominant presence among healthcare workers as well as in the health environment globally (World Health Organization, 2019).

### Reduction of Symptoms Related to Peri- and Post-traumatic Stress

The results show that in both the first and second wave pre-treatment values were significantly higher than post-treatment values for all scales of the IES-R.

As compared to the first wave, in the second wave, healthcare workers showed, both pre- and post-treatment, significantly higher scores in all clusters of the IES-R (total and subscales) although they reached statistical significance only in the subscale hyperarousal pre-treatment. Healthcare workers treated at the beginning of the second wave showed treatment signs of heightened hypervigilance, irritability, memory and concentration problems, sleep disturbances, and exaggerated startle responses. This probably was due to the stress accumulated during the first and second waves. Indeed, the health workers who did not have psychological support in the first wave had a higher level of post-traumatic responses. This is in line with the fact that if there is no specific psychological support in the acute phase of traumatization (which went from the end of February to May), subjects are more vulnerable to other events like the second wave that can reactivate and enhance stress reactions. During the treatment, marked physiological reactions were observed in response to triggers reactivating the traumas experienced in the first wave and not processed (images of the dirty and clean area, separation cloths, protective garments, movement from COVID-19 free to COVID-19 or critical area wards, and disturbing and traumatic images related to the relationship with patients and death events). The results are in line with studies on work-related stress (De Mei et al., 2020) and the risk of burnout (Giusti et al., 2020) among healthcare workers.

A statistically significant reduction of the post-treatment values of the IES-R in both emergency waves confirmed the effectiveness of EMDR therapy in alleviating the emotional suffering associated with the repetition of reliving memories of the traumatic experience through images, sounds (such as the “noise” of CPAP), and physical sensations (dressing), restoring the natural processing, and the feeling of being in control.

Healthcare workers treated early in the first wave presented on average a mean total post-treatment IES-R value below the threshold (<33) for the risk of developing PTSD, while healthcare workers treated in the second wave remained on average a mean total post-treatment IES-R value slightly above the threshold. Moreover, the percentage of healthcare workers losing post-treatment the risk to develop PTSD was higher in the first wave as compared to the second wave, suggesting that a longer exposure without a prompt therapeutic intervention caused in the latter group a more severe and settled COVID-19-related trauma. Although the COVID-19 emergence is unprecedented, the results confirm the importance of early intervention with EMDR as it was found in populations exposed to natural disasters (Saltini et al., 2018).

### Clinical Improvement

The results show that there are no significant differences between the first and second waves with regard to the effect of the treatment with EMDR-IGTP. This protocol is equally effective regardless of time. This finding is in line with theoretical models (Shapiro, 1995), which emphasize that a difference in results is not expected in relation to, for example, the chronicity of the disorder. Furthermore, contrary to what we would expect, the “provenance” covariate does not have a significant effect on either the pre-treatment condition or the pre- and post-treatment total IES-R delta in both the first and second wave. Healthcare workers in hospitals were all equally exposed to the health emergency regardless of the department in which they worked (critical care area, converted COVID wards, or others). This fact can be explained with reference to the particular conditions dictated by this emergency, such as the prolonged isolation experienced by healthcare workers from the rest of the community who, especially at the beginning of the various emergency waves, found themselves spending a significant time inside hospitals, in which access was prohibited to relatives and outsiders, sharing a condition of continuous exposure to a lot of patients. Furthermore, all these were exacerbated by the unreadiness to deal with an unprecedented emergency. This finding is in line with the studies performed after the SARS pandemic (Chan and Huak, 2004; Lee et al., 2007) that have shown significant results regarding the presence of post-traumatic symptoms among healthcare workers related to the lack of preparation to deal with the emergency. Finally, this study shows that the treatment with EMDR-IGTP produced rapid changes in reducing psychological distress regardless of the duration of the exposure of healthcare workers to the COVID-19 outbreak and the department to which they belong. In this perspective, in healthcare workers, EMDR-IGTP was an effective means of reducing emotional suffering, aiding the natural elaboration process as well as the internal control drive. After completing the EMDR sessions, the disturbing memories of the traumatic event are desensitized, losing their negative emotional charge. Changes are fast and the images change in the way they appear. Clients often refer that they see it as more distant and less vivid. Intrusive thoughts are less frequent or do not present themselves anymore. The experience is more adaptive from the therapeutical point of view and at the same time, the emotions and physical sensations are less intense (Jarero et al., 2006, 2008; Shapiro, 2018). Processing the traumatic experience through desensitization and cognitive restructuring has allowed healthcare workers to modify the self-negative judgment integrating emotions appropriate to the current situation as well as reducing or erasing the relative physical response allowing them to adopt more adaptive behaviors.

To be affected by COVID-19 had no impact on the level of clinical improvement or severity for healthcare workers treated in the first wave and re-tested at the beginning of the second wave. Instead, we found significant differences in the personnel treated for the first time in the second wave if we consider the differences between pre- and post-treatment in the case of healthcare workers who were COVID-19 positive compared to those who were not ill. The scores of the pre-treatment variables were higher to be expected in those who fell ill with COVID-19 although only the values of the subscale avoidance of the IES-R reached statistical significance. Those who fell ill with COVID-19 at the beginning of treatment showed a marked avoidance of stimuli associated with the traumatic event that we can explain with reference to the aspects of vicarious and direct traumatization to which healthcare workers were exposed in this health emergency (Invernizzi et al., 2020a).

### Maintenance of the Achieved Outcome Over Time

What happens to healthcare workers treated in the first wave and re-tested at the start of the second wave? Did they maintain or “lose” the benefits of treatment over time when they face the second wave?

The results of this study showed that, following the treatment with EMDR-IGTP, these healthcare workers maintained the positive changes over time despite prolonged exposure to the emergency and the possibility of retraumatization at the onset of a new emergency phase, showing themselves to be less vulnerable and more resilient than healthcare workers not treated early in the first wave.

This confirms the importance of having offered early and timely treatment to healthcare workers in an emergent situation that occurred in waves. Furthermore, a recent publication has highlighted the cost-effectiveness of EMDR therapy in the treatment of PTSDs, having brief therapies a higher level of efficacy (Mavranezouli et al., 2020).

### Limitations

The main limitation of this observational study is represented by the absence of a psychological evaluation in a non-treated population from the very beginning of this study, but the exceptional characteristics of the health emergency imposed the need to treat promptly and urgently any member of the personnel who found himself working in hospitals risking his physical and psychological health. Such a priority resulted in a professional and an ethical choice made in an exceptional hospital context in which we found ourselves working.

However, healthcare workers evaluated only at the beginning of the second wave can be a control group to which compare the effect of treatment.

## Conclusion

The COVID-19 public health emergency had a significant impact on the well-being of healthcare workers who were working in hospitals and mandated the need for mental health protection, support, and treatment. This study demonstrated that early psychological support interventions with the group EMDR-IGTP for this population had a positive effect in both first and second emergency waves to significantly decrease the consequences of acute stress.

Moreover, the change perceived following the treatment was maintained over time, in the presence of new epidemic phases, demonstrating the possibility of strengthening the resilience of healthcare workers and mitigating their vulnerability. All these confirm that working with EMDR in emergencies not only promotes faster recovery but also offers protective factors for re-exposure to other stressful events.

The need to explore new frontiers to integrate mental health into all public health response activities to COVID-19 has forced therapists to adapt protocols and also to provide protection from the risk of vicarious and direct traumatization. We observed that the EMDR-IGTP protocol being so effective can protect psychologists and clinicians from this kind of traumatization. Nonetheless, we never lost our commitment as psychologists, planning and treating all healthcare workers who requested it; they represented a vulnerable group exposed to the development of post-traumatic stress reactions, but at the same time they were a fundamental resource in our hospitals during the COVID-19 outbreak.

## Data Availability Statement

The raw data supporting the conclusions of this article will be made available by the authors, without undue reservation.

## Ethics Statement

This study was approved by the Ethics Committee ATS Brianza with the acronym HOPE (Healthcare wOrkers Psichology Emdr). The participants provided their written informed consent to participate in this study.

## Author Contributions

EFo and RI contributed to the conception and design of this study. EFr was the principal investigator of this research (PI). RI was a co-investigator (co-PI). RI and EFo organized the database. MP performed the statistical analysis. EFo, RI, IF, and MP were responsible for drafting the manuscript. All authors critically revised it for important clinical content, gave final approval to the finished manuscript, and agreed to be accountable for all aspects of the work.

## Conflict of Interest

The authors declare that the research was conducted in the absence of any commercial or financial relationships that could be construed as a potential conflict of interest.

## Publisher’s Note

All claims expressed in this article are solely those of the authors and do not necessarily represent those of their affiliated organizations, or those of the publisher, the editors and the reviewers. Any product that may be evaluated in this article, or claim that may be made by its manufacturer, is not guaranteed or endorsed by the publisher.
